# The Effect of Different Cleaning Methods on Protein Deposition and Optical Characteristics of Orthokeratology Lenses

**DOI:** 10.3390/polym13244318

**Published:** 2021-12-09

**Authors:** Chen-Ying Su, Lung-Kun Yeh, Yi-Fei Tsao, Wen-Pin Lin, Chiun-Ho Hou, Hsueh-Fang Huang, Chi-Chun Lai, Hsu-Wei Fang

**Affiliations:** 1 Department of Chemical Engineering and Biotechnology, National Taipei University of Technology, 1, Sec. 3, Zhongxiao E. Rd., Taipei 10608, Taiwan; chenying.su@ntut.edu.tw (C.-Y.S.); susan84103086@gmail.com (Y.-F.T.); 2 Department of Ophthalmology, Chang Gung Memorial Hospital, Linkou, No. 5, Fuxing St., Taoyuan 333, Taiwan; yehlungkun@gmail.com (L.-K.Y.); chiunho@cgmh.org.tw (C.-H.H.); chichun.lai@gmail.com (C.-C.L.); 3 College of Medicine, Chang Gung University, No. 259, Wenhua 1st Rd., Taoyuan 333, Taiwan; 4 Research and Development Center, Brighten Optix Co., 6F-1, No. 150, Sec. 4, Chengde Rd., Shilin Dist., Taipei 111, Taiwan; benson.lin@brightenoptix.com (W.-P.L.); sherry.huang@brightenoptix.com (H.-F.H.); 5 Department of Optometry, University of Kang Ning, No. 137, Alley 75, Sec. 3, Kang Ning Road, Neihu District, Taipei 11485, Taiwan; 6 Institute of Biomedical Engineering and Nanomedicine, National Health Research Institutes, No. 35, Keyan Road, Zhunan Town, Miaoli County 35053, Taiwan

**Keywords:** orthokeratology lens, protein deposition, optical characteristics, rubbing

## Abstract

Orthokeratology lenses are commonly used for myopia control, especially in children. Tear lipids and proteins are immediately adsorbed when the lens is put on the cornea, and protein deposition may cause discomfort or infection. Therefore, we established an in vitro protein deposition analysis by mimicking the current cleaning methods for orthokeratology lens wearers for both short-term and long-term period. The results showed that the amounts of tear proteins accumulated daily and achieved a balance after 14 days when the lens was rubbed to clean or not. Protein deposition also affected the optical characteristics of the lens regardless of cleaning methods. Our results provided an in vitro analysis for protein deposition on the lens, and they may provide a potential effective method for developing care solutions or methods that can more effectively remove tear components from orthokeratology lenses.

## 1. Introduction

Orthokeratology (ortho-k) lenses have been used for affecting vision since 1945, when the corneal contact lens was made with plastic [1]. Modern ortho-k lens can be worn overnight to reshape the anterior cornea by the designs of reverse geometry; thus, patients with myopia do not need to wear glasses for vision correction during the daytime [2]. The safety of wearing ortho-k lenses has been monitored by several long-term clinical follow-ups, and complications still arise, although the majority was not immediate adverse events [3]. The major complications are microbial keratitis and superficial corneal staining, while microbial keratitis is a potentially vision-threating complication [4]. The possible cause of microbial keratitis might due to lack of eye movements which usually help to disrupt the bacterial glycocalyx, resulting in less spreading of lysozyme over the surface of eye tissue and making eyes being more susceptible to the infection [5]. In addition, microbial keratitis might be non-compliance, including inappropriate lens care and wearing continuously despite significant discomfort [6,7]. Indeed, tear proteins and lipids are immediately deposited on the surface of the lens when a contact lens is put into the eye. Tear proteins are also easily accumulated on the lens and may cause immune reactions if lenses are not being cleaned completely [8,9].

Ortho-k lenses can be reused for at least one year; thus, lens care is a more important and critical compared with soft contact lenses that are made as disposable daily. It has been shown that cleaning rigid contact lenses with lens care solutions without finger rubbing was not an effective method for removing stubborn materials (such as mascara and hand cream) from the lens [10]. Rigid contact lens multipurpose care solutions, hydrogen peroxide, and povidone–iodine are all advised for cleaning ortho-k lenses, but the related studies only focused on disinfecting microorganisms [7]. The studies about removing tear components from ortho-k lenses have not yet been investigated intensively. In addition, it is also possible that an accumulation of tear components may affect the reverse geometry of ortho-k lens, resulting in ineffectiveness in flattering the cornea. The relationship between tear components accumulation and the optical characteristics of ortho-k lenses is still unclear.

In this study, we first investigated protein deposition in the absence or presence of tear lipids. Two tear proteins were analyzed here: the lysozyme and albumin. Lysozyme is the most abundant tear protein, while the concentration of albumin increases when the eye is closed or when wearing ortho-k lenses [11,12]. Protein adsorption amounts on ortho-k lenses were analyzed by cleaning with or without finger rubbing. Both short-term and long-term protein deposition analyses were conducted, and the accumulated protein amounts were quantified daily. In addition, the optical characteristics of ortho-k lens after a long-term protein deposition procedure were also investigated.

## 2. Materials and Methods

### 2.1. Orthokeratology Lenses, Contact Lens Care Solution, and Artificial Tear solution

The material of ortho-k lenses used in the study was Boston XO^TM^, and the material generic name was Hexafocon A (Brighten Optix Co., Taipei, Taiwan). The contact lens care solution used for cleaning and rinsing was Menicon Care Plus multipurpose solution for all rigid gas permeable lenses (Menicon, Nagoya, Japan). The preparation of artificial tear solution has been published previously [13]. In general, a complex of salt solution was made according to the concentration listed in Table 1 [13]. The pH value of a complex of salt solution was adjusted to 7.4 and was stored at room temperature for 3 or more days. The 2000 times lipid stock solution was made in a solution of 1 hexane:1 ether, and the stock concentration was listed in Table 1. Then, 250 μL of 2000 times lipid stock solution was added into a complex of salt solution, and the mixed solution was placed into an ultra-sonic bath at 37 °C. The mixed solution was sonicated at 90 W and purged with nitrogen gas until the lipid stock solution was fully incorporated, and the odor of hexane and ether was cleared out. Then, either lysozyme or albumin was added into the mixed solution containing salts and lipids for the further experiments. All the components of salt, lipid, and protein were purchased from Sigma-Aldeich (St. Louis, MO, USA).

### 2.2. Short-Term Protein Deposition Analysis

**Figure 1:**
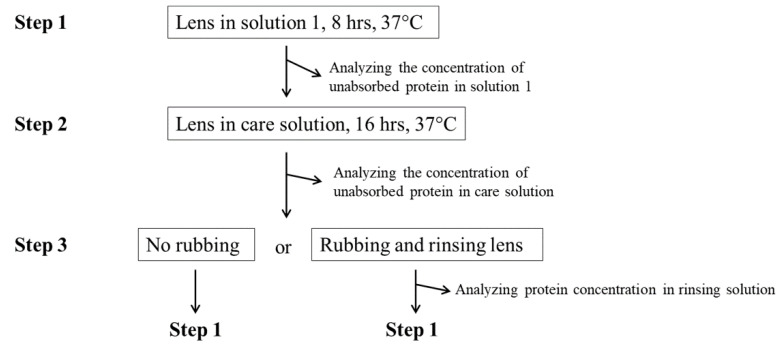
The procedure of protein deposition analysis. One completed cycle is from step 1 to step 3.

The ortho-k lens was treated by an oxygen plasma system (Force State OP300, PFS Co., Ltd., Taipei, Taiwan) at 100 mTorr, 80 W, 10 standard cubic centimeter per minute (Sccm) for 120 s before the experiment, in order to improve the hydrophobicity. The orthokeratology lens was placed in 2 mL of solution 1, which was artificial tear solution containing either 2.0 mg/mL lysozyme or 0.2 mg/mL albumin, and incubated at 37 °C for 8 h (Step 1 in Figure 1). Then, the immersed lens was placed in 2 mL of care solution at 37 °C for 16 h (Step 2 in Figure 1). Finally, the ortho-k lens was not cleaned (no rubbing) by care solution and was placed into a new artificial tear solution. The other group was cleaned (rubbing), rinsed with fingers in gloves by 50 μL of care solution, and placed into a new artificial tear solution (Step 3 in Figure 1). A completed cycle (step 1 to 3) was repeated 5 times (5 days), and the protein concentrations in each solution were analyzed.

The Bio-Rad DC protein assay (Bio-Rad, Hercules, CA, USA) was used for measuring the amount of lysozyme or albumin in each solution [14]. The optical density (OD) value was obtained by an Enzyme-Linked Immunosorbent Assay (ELISA) reader with a wavelength of 280 nm. The deposited protein concentration on the orthokeratology lens after 3 steps on each was calculated as follows: (the original protein concentration)–(protein concentration in solution 1)–(protein concentration in care solution) for the no rubbing group. For the rubbing group, the deposited protein concentration was calculated as follows: (the original protein concentration)–(protein concentration in solution 1)–(protein concentration in care solution)–(protein concentration in rinsing solution). Five independent lenses were tested for each condition.

### 2.3. Long-Term Lysosomal Deposition Assay

In addition to a short-term protein deposition analysis, a long-term lysosomal deposition assay was also investigated. Ortho-k lenses were placed in artificial tear solution containing 2.0 mg/mL of lysozyme, and the procedure was same as shown in Figure 1. Thirty cycles (30 days) were conducted for a long-term analysis.

### 2.4. Optical Characteristics of Orthokeratology Lens

Light microscope VMS3020 (Shanghai Jingmi, Shanghai, China) was used for observing the surface of ortho-k lenses after protein deposition analysis. The pictures were taken with 10× objective and 10× ocular lens magnification. The base curve, central thickness, power and transmission of vision light (VIS), ultraviolet A (UVA), and ultraviolet B (UVB) light of ortho-k lenses were measured by CG Auto II (Neitz Instruments Co., Ltd., Tokyo, Japan), Contest plus (Rotlex, Omer, Israel), and UV/VIS Spectrophotometer (Kingtech Scientific Co., Ltd., Taipei, Taiwan). The water contact angle of lenses was measured by a MagicDroplet Contact Angle Meter Model 100SB (Sindatek Instruments Co., Ltd., Taipei, Taiwan). According to the standard ISO (International Standardization Organization) 18369-2 [15], the difference of base curve should be ±0.02 mm, central thickness should be ±0.02 mm, and power should be ±0.25 degree after any treatments. Otherwise, the measured value was considered as out of tolerance.

### 2.5. Statistical Analysis

The 2-tailed t-test was assessed in order to compare differences in lysozyme or albumin deposition amounts between two different conditions, such as comparison of protein deposition concentration between no rubbing and rubbing on day 1. A value of *p* < 0.05 was considered significant.

## 3. Results

### 3.1. Proteins Are Absorbed Increasingly in the Presence of Lipids

**Figure 2:**
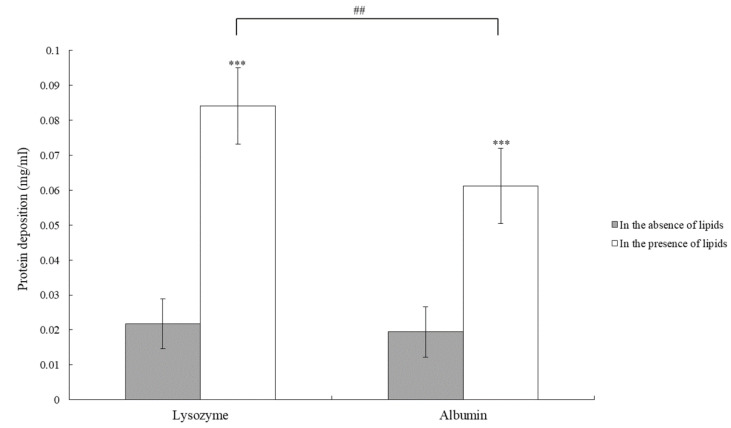
The concentration of protein deposition on the ortho-K lens in the absence of lipids (gray bars) or in the presence of lipids (white bars). *** *p* < 0.001 when comparing protein deposition amount in the absence of lipids versus in the presence of lipids. ## *p* < 0.01 when comparing the deposition amount of lysozyme versus albumin. Error bars represented standard deviation.

In order to mimic the condition of wearing ortho-k lenses, the lens was placed in artificial tear solution containing salts, lipids, and proteins. In addition, ortho-k lenses were placed in solution containing only salts and proteins to compare the amounts of protein deposition. The result showed that in the presence of lipids, the amounts of lysozyme or albumin were dramatically increased compared with the amounts in the absence of lipids (Figure 2).

### 3.2. Rubbing the Lens Effectively Removes Absorbed Proteins from the Lens

**Figure 3:**
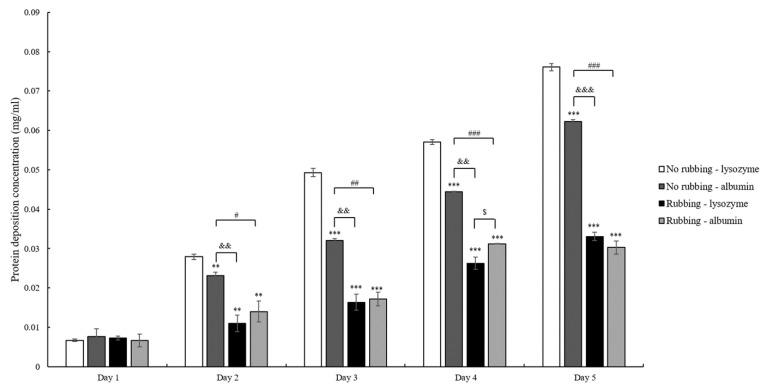
The concentration of protein deposition is accumulated when the lens is not rubbed (white or dark gray bars) or is rubbed (black or light gray bars) for cleaning. ** *p* < 0.01 and *** *p* < 0.001 when comparing protein deposition amount with lysozyme concentration on the lens without rubbing. && *p* < 0.01 and &&& *p* < 0.001 when comparing albumin deposition on no ribbing lens versus lysozyme deposition on rubbing lens. # *p* < 0.05, ## *p* < 0.01, and ### *p* < 0.001 when comparing albumin deposition on the lens without rubbing versus with rubbing. $ *p* < 0.05 when comparing lysozyme versus albumin deposition on the lens with rubbing. Error bars represented standard deviation.

Ortho-k lens wearers are advised to store lenses into care solution after wearing and then rub lenses to clean before putting back into the eye (Figure 1). However, many wearers may not rub the lenses to avoid breaking the lens. Then, the protein deposition on the lens with and without rubbing was investigated. The result demonstrated that protein deposition was increased daily regardless of no rubbing or rubbing (Figure 3). It was obvious that the amount of protein deposition on the lens without rubbing was more than the amount with rubbing. In addition, the amount of lysozyme deposition was significantly more than the amount of albumin when the lens was not rubbed to clean. The amount of lysozyme and albumin deposition was not similar when the lens was rubbed (Figure 3).

### 3.3. Lysozyme Deposition on the Lens Tends to Be Stable after 14 Days

**Figure 4:**
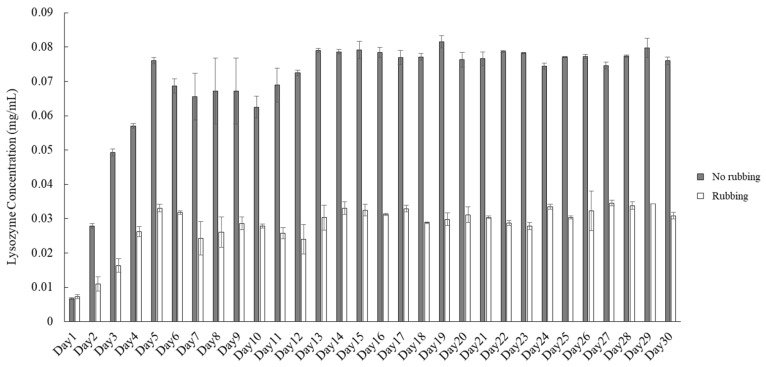
Deposited lysozyme concentrations are measured after the lens after one cycle of procedure. The lens is either not rubbed (gray bars) or rubbed (white bars) at the end of one cycle. The difference is statistically significant (*p* < 0.001) when comparing lysozyme deposition concentration on no rubbing versus rubbing lenses on the same day. Error bars represented standard deviation.

To understand the longer effect of cleaning methods on protein deposition, the procedure of protein deposition (Figure 1) was repeated for 30 days. The amount of lysozyme deposition was greatly reduced if the lens was rubbed compared with not rubbed (Figure 4). Lysozyme deposition was increased greatly during the first few days of the procedure no matter whether the lens was rubbed or not, which was similar with the observation when the procedure was repeated for 5 days (Figure 3). Although the concentration of lysozyme deposition was not increased dramatically after day 13, the current cleaning methods were also unable to remove lysozyme effectively once it was adsorbed onto the lens.

### 3.4. Optical Characteristics of Orthokeratology Lenses Changed after Long-Term of Lysozyme Deposition

**Figure 5:**
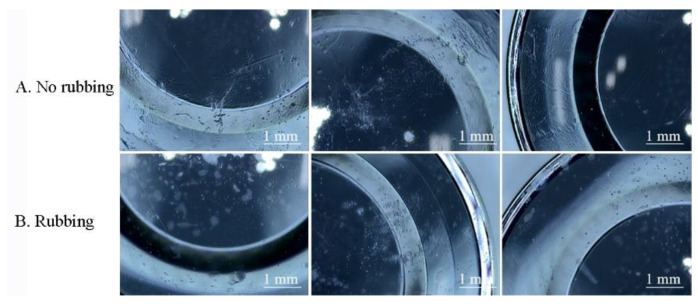
Pictures of lens surface after 30 cycles of protein deposition procedure when the lens was not rubbed (**A**) or rubbed (**B**) at the end of each cycle.

Then, the optical characteristics of ortho-k lenses were investigated after 30 cycles of protein deposition procedure. There was contamination accumulated on the surface of the lens when the lens was not rubbed (Figure 5A) or rubbed (Figure 5B) with care solution. In addition, the power (PW) of each lens changed after 30 cycles of procedure (Table 2) as well as the transmission of VIS, UVA, and UVB (Table 3). The base curve of item 1 and the central thickness of item 3 in rubbing group were out of tolerance. The contact angle was also tested for each lens after 30 days, and the result showed that the contact angle was lower if the lens was not rubbed at the end of each cycle (Table 2).

## 4. Discussion

We analyzed both short-term and long-term protein deposition on ortho-k lenses and the effect of protein deposition on optical characteristics of ortho-k lenses. We first investigated the effect of tear lipids on protein deposition, and the result demonstrated that the amounts of protein deposition were affected by the presence of lipids. Indeed, it has been shown that protein deposition increased dramatically in the presence of lipids on rigid gas-permeable contact lenses [16]. The previous study suggested that the hydrophobic nature of ortho-k lenses may attract the hydrophobic sites of lipids, resulting in the exposure of hydrophilic sites to attract protein binding [16]. Then, we next mimicked the possible cleaning methods that are used by ortho-k lens wearers. Although the wearers are advised to rub and rinse ortho-k lenses, many wearers start to skip rubbing because of being lazy or anxious about breaking expensive lenses [7,10]. The results showed that the amount of protein deposited on the lens was higher when the rubbing step was skipped, and the difference between rubbing and non-rubbing could be observed during the first few days (Figure 2 and Figure 3). Therefore, rubbing the lens should continuously be advised to ortho-k lens wearers in order to remove deposited tear proteins.

A short-term protein deposition analysis showed that the deposition concentration of lysozyme and albumin was similar when the lens was rubbed to clean, whereas the concentration of lysozyme deposition was high when the rubbing step was skipped (Figure 2). The initial concentration of lysozyme was 10 times higher than albumin; thus, it was not surprising when lysozyme was adsorbed onto the lens more than albumin in the non-rubbing group. In addition, the surface of the ortho-k lenses is negatively charged [16]. The isoelectric point (pI) of albumin and lysozyme is 5.16 and 11.4, respectively [17,18]. Therefore, lysozyme is attracted and bound to the surface of the ortho-k lens more easily than albumin, resulting in more lysozyme deposition than albumin when the lens was not rubbed to clean. In contrast, lysozyme seemed to be removed more easily than albumin when the lens was rubbed to clean. Protein deposition onto the contact lens is not only affected by the charge of protein but other factors including the materials used for the contact lens, water content, pore size, protein size, protein structure, hydrophobicity, etc. can also play a vital role [17]. When the lens was rubbed, both lipids and proteins should be removed. We observed that the contact angle of the lens without rubbing was smaller than the lens with rubbing, suggesting that the lens with rubbing could maintain its hydrophobicity (Table 2). It has been shown that albumin was more easily denatured on the hydrophobic surface than on the hydrophilic surface [19], and it is possible that albumin was denatured after rubbing, resulting in it being difficult to be removed, and the final deposition concentration was similar to lysozyme. Whether albumin was more difficult to be removed from the ortho-k lens than lysozyme will require further investigation.

The result of a long-term protein deposition analysis demonstrated that the concentration of lysozyme deposition was saturated after 14 days whether the lens was rubbed to clean or not, suggesting that a balance between lysozyme adsorption and desorption was achieved. The assay we used for measuring protein concentration was an indirect method, because protein was not directly extracted from the lens. However, this indirect method allowed us to observe the daily accumulation of tear proteins, and the investigation could repeat for a long term in order to mimic the clinical observation. The result also suggested that the lens could not be completely cleaned by the current cleaning method for ortho-k lens wearers (Figure 5). Hydrogen peroxide and povidone–iodine can also be used for cleaning ortho-k lenses, especially for their effective anti-microbial activity [7]. Hydrogen peroxide has been shown that it can remove lipids or proteins on contact lenses when combining with surfactants or catalytic discs [20,21]. However, hydrogen peroxide cannot remove all the deposition on the lens, indicating that the cleaning ability of current commercial care solutions can still be improved.

In addition, lysozyme deposition changed the optical characteristics of ortho-k lenses, especially the power and the transmission of VIS, UVA, and UVB (Table 2 and Table 3). The ortho-k lens utilizes the reverse geometry to provide hydraulic forces in post-lens tear film and causes stresses across the corneal epithelium, resulting in the reshaping of cornea and slowing the progression of myopia [22]. Therefore, the reduction of power and transmission of lights observed in the ortho-k lenses after a long-term lysosomal deposition might suggest that the reverse geometry design of the lens might be altered. We speculated that protein deposition on ortho-k lenses might not only increase the risk of infection but also reduce the effectiveness of myopia control. However, this possibility has not yet been observed clinically. The further clinical investigation should be analyzed to understand the relationship between tear protein deposition and myopia control of ortho-k lenses.

## 5. Conclusions

The current study demonstrated that tear proteins were more easily adsorbed on the surface of hydrophobic ortho-k lenses in the presence of tear lipids. Both short-term and long-term protein deposition analysis showed that proteins accumulated on the ortho-k lenses, and rubbing could remove significantly more adsorbed proteins than non-rubbing. Lysozyme deposition for a long term affected the optical characteristics of ortho-k lenses, and whether the changes of optical parameters affect the function of myopia control for ortho-k lenses will require clinical investigation. We mimicked clinical methods for cleaning ortho-k lenses, and our results provided in vitro evidence for tear protein accumulation on the lens that may increase risk for infection subsequently.

## Figures and Tables

**Table 1:** The concentration of each component in artificial tear solution.

Category	Component	Stock Concentration (mg/mL)	Final Concentration in Artificial Tear Solution (mg/mL)
Complex of salt solution	Sodium chloride	N.A.	5.26
	Potassium chloride		1.19
	Sodium citrate		0.44
	Glucose		0.036
	Urea		0.072
	Calcium chloride		0.07
	Sodium carbonate		1.27
	Potassium hydrogen carbonate		0.30
	Sodium phosphate dibasic		3.41
	Hydrochloric acid		0.94
	ProClin 300		200 μL/liter of solution
Lipid stock solution	Oleic acid	3.6	0.0018
	Oleic acid methyl ester	24.0	0.012
	Triolein	32.0	0.016
	Cholesterol	3.6	0.0018
	Cholesteryl oleate	48.0	0.024
	Phosphatidylcholine	1.0	0.0005
Protein	Lysozyme	N.A.	2.0
	Albumin		0.2

N.A.: Non applicable.

**Table 2:** Optical specifications of each tested lens before and after 30 cycles of protein deposition procedure.

Cleaning Method	Item	Before			After			
		BC (mm)	CT (mm)	PW (degree)	BC (mm)	CT (mm)	PW (degree)	Contact Angle (°)
No rubbing	1	8.60	0.23	+0.69	8.60	0.23	+0.66	15.09
	2	8.73	0.22	+0.77	8.72	0.22	+0.71	23.71
	3	9.11	0.23	+1.10	9.10	0.23	+1.11	13.02
Rubbing	1	9.01	0.24	+0.93	8.92 #	0.24	+0.74	80.29
	2	8.57	0.23	+0.76	8.57	0.24	+0.76	64.56
	3	8.71	0.21	−0.62	8.71	0.24 ^#^	−0.48	52.32

BC: base curve; CT: central thickness; PW: power. # The measured data were out of tolerance.

**Table 3:** Optical transmission of each tested lens before and after 30 cycles of protein deposition procedure.

Cleaning Method	Item	Before			After		
		VIS	UVA	UVB	VIS	UVA	UVB
No Rubbing	1	83.85	12.63	0.734	85.18	12.86	0.779
	2	88.06	13.96	1.061	83.26	12.89	0.909
	3	82.98	12.24	0.677	76.69	11.52	0.785
Rubbing	1	87.86	13.55	0.831	78.19	10.74	0.523
	2	84.99	12.37	0.629	83.41	12.26	0.675
	3	84.12	13.44	0.987	83.73	13.23	1.014

VIS: vision light; UVA: ultraviolet A light; UVB: ultraviolet B light.

## Data Availability

The data presented in this study are available on request from the corresponding author.
